# Isolated Lunate Fracture in a Child: A Case Report

**DOI:** 10.1016/j.jhsg.2025.03.003

**Published:** 2025-05-21

**Authors:** Lydian Aliene Huisman, Mike Rüttermann

**Affiliations:** ∗ Department of Surgery, University Medical Center Groningen, Groningen, the Netherlands; † Department of Plastic Surgery, University Medical Center Groningen, Groningen, the Netherlands

**Keywords:** Avascular necrosis, Carpal fracture, children, lunate

## Abstract

Isolated lunate fractures in children are exceptionally uncommon. We present a case of a 6-year-old girl with an isolated, displaced lunate fracture after a fall who was treated with semi-open reduction with K-wires, resulting in complete functional recovery. This approach may reduce the risk of avascular necrosis (Kienböck’s disease) of the lunate.

Although children's hands and wrists are exposed to many falls, carpal injuries in children are rare because the carpus is predominantly cartilaginous. Considerable force is required to cause carpal fractures. Lunate fractures often are associated with ligament injuries, other carpal fractures and perilunate dislocations.^1^ Isolated lunate fractures in children are rare; literature on the subject is limited. This report was written according to the Case Report guidelines. Written informed consent was obtained from the patient’s parents for publication of this case report and accompanying images.

## Case Report

**Figure 1:**
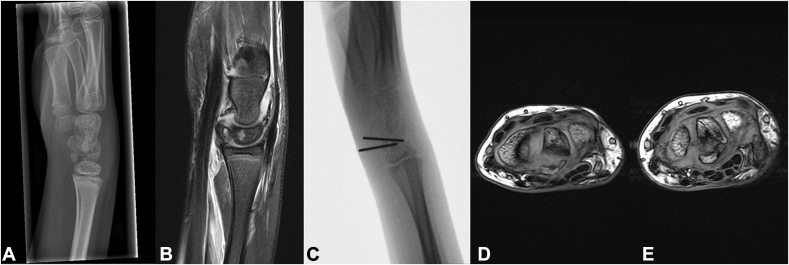
**A** Wrist radiograph one day after injury of the right wrist, showing a displaced lunate fracture. **B** T2-weighted MRI of the right wrist one day after injury, revealing the lunate fracture but no additional ligament injury. **C** Intraoperative lateral view after placing two K-wires to fix the fracture fragments. **D** Approximately 16 months after injury, T1-weighted MRI shows no enhancement of the lunate, possibly indicating inadequate blood perfusion. **E** At 28 months after injury, T1-weighted MRI reveals no consolidation yet of the lunate fragments.

A 6-year-old girl presented one day after a fall on the playground with her wrist in palmar flexion. On physical examination, palmar and dorsal flexion of the wrist was painful but unrestricted. She had dorsal tenderness over the lunate. A wrist radiograph showed a transverse lunate fracture, in the sagittal plane, with two large fragments and a 3 mm diastasis (Fig. 1A), and a small chip between the lunate and triquetrum. Subsequent magnetic resonance imaging (MRI) confirmed this (Fig. 1B) and showed no additional injury to the scapholunate or lunotriquetral ligaments. The smaller fragment also appeared to originate from the lunate and no other fractures were identified.

An attempt at closed reduction under sedation was not successful. Therefore, semi-open reduction and fixation was performed. The carpal tunnel was opened via an anterior approach. With radial retraction of the flexor tendons and median nerve, the two large lunate fragments were repositioned under fluoroscopy using a 1.0 mm K-wire without opening the capsule. Additionally, a divergent 1.0 mm K-wire was placed to fix the repositioned lunate (Fig. 1C). The wrist was immobilized in an intrinsic-plus position until the K-wires were removed at three weeks. A removable splint was fitted for a further three weeks, after which she was able to regain wrist motion under the guidance of a hand therapist. Four months after K-wire removal, palmar and dorsal wrist flexion was limited to 50° (50°–0°–50°), as opposed to the left wrist (70°–0°–70°). Radial and ulnar deviation was unrestricted in both wrists (15°–0°–30°). Wrist radiographs showed in-line carpalia but no complete fracture consolidation.

At 16 months after the initial injury, MRI showed the absence of definitive blood flow to the lunate. However, there was no discernible deterioration in the morphologic integrity of the lunate (Fig. 1D). Clinically, she had no functional complaints, with full active range of motion of the wrist (palmar and dorsal wrist flexion 70°–0°–70°; radial and ulnar deviation 15°–0°–30°), fingers and thumb. A further six months later, she still had full active range of motion and full weightbearing tolerance without any functional complaints. At 28 months after the injury, MRI showed no further fragmentation or osteonecrosis, although the lunate fragments still were not consolidated (Fig. 1E). The patient still had no functional complaints and full active range of motion of the wrist and hand.

## Discussion

Lunate fractures in children are extremely rare; in adults, they account for approximately 4% of carpal injuries. One case has been reported of a child with an isolated lunate fracture, requiring open reduction and osteosynthesis. Unfortunately, the lunate became necrotic.^2^ A serious complication of displaced lunate fractures is avascular necrosis of the lunate (Kienböck’s disease) because of impaired arterial perfusion, which can lead to wrist instability and osteoarthritis. Early surgical intervention with anatomic reduction followed by fixation is thought to reduce this risk.^3^

Semi-open reduction with the capsule intact improves, or at least does not further reduce, the blood supply to the already injured lunate. This may reduce the risk of avascular necrosis and improve the active range of motion compared to open reduction and fixation. Given the young age of our patient, screw fixation in the still cartilaginous lunate would not have provided stable fixation, notwithstanding the problems with further growth.

Adequate early diagnosis and management of lunate fractures is necessary to achieve satisfactory function and reduce the risk of complications.

## Conflicts of Interest

No benefits in any form have been received or will be received related directly to this article.
